# CHILDHOOD ADVERSITY IMPACTS PERSONALITY DEVELOPMENT ACROSS ADULTHOOD

**DOI:** 10.1093/geroni/igac059.2880

**Published:** 2022-12-20

**Authors:** Sarah Miller, Meredith Willard, Jacob Alderson, Nicholas Turiano

**Affiliations:** West Virginia University, Morgantown, West Virginia, United States; West Virginia University, Morgantown, West Virginia, United States; West Virginia University, Morgantown, West Virginia, United States; West Virginia University, Morgantown, West Virginia, United States

## Abstract

Throughout adulthood, people’s personalities typically change favorably such that agreeableness and conscientiousness increase, and neuroticism decreases. However, the impact of early life adversity on these changes in personality has yet to be examined. We utilized data from 6,382 community dwelling adults (ages 25–75) that completed 3-waves of the Midlife Development in the U.S. Study (MIDUS) in 1995–96, 2005–06, and 2013–14. Early life adversity was computed using 16 retrospective items assessing emotional and physical abuse, socioeconomic disadvantage, familial instability, and early-life poor health. Personality was assessed with the Big 5 MIDI measure. Latent growth curves confirmed there was significant variability in change over the ~20 year follow-up (all RMSEAS < .06; and CFI/TLI > .96). More specifically, higher levels of early adversity predicted higher initial levels of neuroticism, and lower levels of conscientiousness and extraversion. Additionally, higher adversity predicted a steeper decrease in neuroticism across 20-years (all p’s < .05). Examination of specific adversity types revealed that emotional abuse and having poor health at a young age were especially detrimental to personality development. This research provides evidence that high levels of adverse experiences in childhood predict unfavorable personality development throughout adulthood. Moreover, this research demonstrates that early life adversity, especially certain types, can have lifelong detrimental effects on development. Interventions for those who have experienced adversity should be implemented as early in life as possible to prevent suboptimal psychological development trajectories. Such interventions could improve life outcomes if personality development is more normalized.

